# More attention on glial cells to have better recovery after spinal cord injury

**DOI:** 10.1016/j.bbrep.2020.100905

**Published:** 2021-01-25

**Authors:** Sajad Hassanzadeh, Maryam Jalessi, Seyed Behnamedin Jameie, Mehdi Khanmohammadi, Zohre Bagher, Zeinab Namjoo, Seyed Mohammad Davachi

**Affiliations:** a Skull Base Research Center, Hazrat Rasoul Hospital, The Five Senses Health Institute, Iran University of Medical Sciences, Tehran, Iran; b Neuroscience Research Center (NRC), Iran University of Medical Sciences, Tehran, Iran; c Department of Medical Basic Sciences, Faculty of Allied Medicine, Iran University of Medical Sciences, Tehran, Iran; d ENT and Head & Neck Research Center and Department, The Five Senses Health Institute, Hazrat Rasoul Akram Hospital, Iran University of Medical Sciences, Tehran, Iran; e Department of Anatomical Sciences, School of Medicine, Ardabil University of Medical Sciences, Ardabil, Iran; f Department of Food Science, College of Agriculture and Life Sciences, Cornell University, Ithaca, NY, USA

**Keywords:** Spinal cord injury, A1 astrocyte, A2 astrocyte, OPCs, M1 and M2 macrophages, CNS, Central Nervous System, SCI, Spinal Cord Injury, OPCs, Oligodendrocytes Progenitor Cells

## Abstract

Functional improvement after spinal cord injury remains an unsolved difficulty. Glial scars, a major component of SCI lesions, are very effective in improving the rate of this recovery. Such scars are a result of complex interaction mechanisms involving three major cells, namely, astrocytes, oligodendrocytes, and microglia. In recent years, scientists have identified two subtypes of reactive astrocytes, namely, A1 astrocytes that induce the rapid death of neurons and oligodendrocytes, and A2 astrocytes that promote neuronal survival. Moreover, recent studies have suggested that the macrophage polarization state is more of a continuum between M1 and M2 macrophages. M1 macrophages that encourage the inflammation process kill their surrounding cells and inhibit cellular proliferation. In contrast, M2 macrophages promote cell proliferation, tissue growth, and regeneration. Furthermore, the ability of oligodendrocyte precursor cells to differentiate into adult oligodendrocytes or even neurons has been reviewed. Here, we first scrutinize recent findings on glial cell subtypes and their beneficial or detrimental effects after spinal cord injury. Second, we discuss how we may be able to help the functional recovery process after injury.

## Highlights

• Reactive astrocytes are divided into two subsets: A1 astrocytes and A2 astrocytes.
• The transformation of A1 to A2 reactive astrocytes can be a beneficial strategy for better repair.
• The NeuroD1 factor can be used to convert most astrocytes into the neurons or the A2 astrocytes.
• Differentiation of oligodendrocytes to their progenitor cells after injury may be useful for better response.
• Increase the ratio of M2/M1 macrophages at the injury site is a potential therapeutic solution.

## 1 Introduction

An injury to the central nervous system (CNS), particularly to the spinal cord, causes irreversible damage. After an injury, however, low degrees of functional improvement has been observed in some patients. The rate of this improvement, although minor, is clearly visible on the cortical map. Furthermore, these changes in plasticity are seen at different levels, including the brainstem, spinal cord, and CNS [2]. Pathological events after spinal cord injury (SCI) include primary and secondary damages. Primary post-SCI damages occur immediately after injury and include demyelination and necrosis, as well as neuronal and axonal loss [3]. Secondary post-SCI damages include persistent demyelination and neuronal loss, edema and nerve ischemia, oxidative stress, inflammatory reactions, and glial scar formation [[4], [5], [6], [7]]. The CNS consists of two types of cells: the neurons, which are responsible for communicating with other neurons and perceiving changes, and the glia, also called glial cells or neuroglia, which are responsible for nourishing, protecting, and supporting the nervous system, as well as removing waste products from the system. Glial cells are a group of cells that are highly involved in the damages incurred after SCI. Many studies have shown that these CNS cells play a key role both in the process of regeneration and functional improvement (beneficial) as well as in accelerating traumatic injuries (harmful). Three main groups of glial cells exist in the CNS which are affected by spinal injuries. The first group is the astrocytes that regulate neurotransmitter and neurovascular dynamics in the CNS. After SCI, these cells turn into reactive astrocytes that cause glial scar formation and eventually restrict plasticity. The second group are microglia, which are responsible for scanning degrees of infection and injury, and increase axonal formation and remyelination in response to SCI, but also develop cytotoxic effects. Lastly, oligodendrocytes and their precursor cells are responsible for supporting the axons as well as accelerating axonal signaling in the CNS. In response to SCI, they differentiate and produce lost myelin, but they are susceptible to death [8].

Our aim in this review is to investigate the effects of these three cell types on post-SCI events and suggest viable ways to assist in the post-SCI recovery process.

### 1.1 Astrocytes

Astrocytes, the most abundant cells in the CNS, are present in both white matter and grey matter. These cells have a wide range of functions in the CNS and play a crucial role in neurophysiology. Astrocytes contribute to synaptogenesis, regulate neurotransmitters, play an essential role in the immune response, promote the expression of extracellular matrix molecules, improve cell migration, and ultimately enhance differentiation and maturation in the CNS [9].

### 1.2 Morphology of astrocytes after SCI

**Fig. 1:**
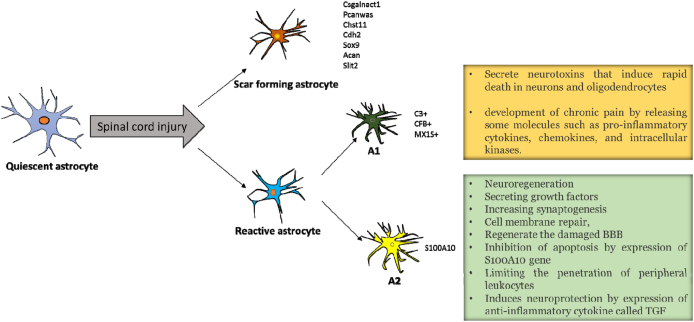
Changes in the morphology and function of quiescent astrocytes after an injury to the spinal cord. Quiescent astrocytes are able to be divided into reactive and scar-forming astrocytes; reactive astrocytes can be further classified into A1 and A2 astrocytes. Each of these cells has its own markers and functions after SCI.

Astrocytes are one of the most crucial cell subtypes in the nervous system which are involved in establishing and maintaining homeostasis during an injury. In various conditions of damage to neural tissue, changes occur in the function, appearance, and gene expression of nerve cells. The post-injury changes in astrocytes are called Astrocytopathy, which is divided into two main categories of functional and phenotype changes. These cells may be inactive, quiescent, active, or reactive [10]. The quiescent astrocytes are present in the normal CNS tissue (Fig. 1). Under conditions of nerve damage or bleeding, existing astrocytes are transformed into several subsets of reactive and scar-forming astrocytes. These cells have morphologically hypertrophy and their appendages increase and expand. In the astrocytes following the formation of reactive or scar-forming types, the expression of markers was also increased, such as GFAB, β-catenin, nestin, and N-cadherin. However, it should be noted that each of these astrocytes also has its own markers, and along with changes in their phenotype can be considered as detectors [[11], [12], [13]]. Reactive astrocyte markers contain matrix metalloproteinase-2 (MMP-2), Plaur, MMP13, Axin2, Nes, and Ctnnb1 gens. The scar-forming astrocyte markers contain Cdh2, Sox9, Csgalnact1, Chst11, Pcanwas, Acan, and Slit2 gens [[11], [12], [13]].

### 1.3 Subtypes of reactive astrocytes

Recently it has been confirmed that reactive astrocytes are divided into two subsets [14,15]: A1 astrocytes, which are harmful and induce death in neurons and oligodendrocytes, and A2 astrocytes, which are beneficial and promote neuronal viability and neuroregeneration. Neuro inflammation-induced A1 astrocytes to secrete neurotoxins that in turn induced rapid death in neurons and oligodendrocytes. The C3, CFB, and MX1S are the genes expressed in type A1 and considered as markers in this type of astrocyte [[14], [16]]. The A1 astrocytes lose many of their normal functions, such as contributing to neuronal viability and growth, inducing fewer and weaker synapses than common astrocytes, and progressive killing of adult neurons. In addition, A1 astrocytes have been associated with a variety of human neurodegenerative diseases such as amyotrophic lateral sclerosis and Alzheimer's disease [17,18]. Studies show that deleterious post-injury effects seen due to reactive astrocytes can be attributed to A1 reactive astrocytes. For instance, in Alzheimer's disease, approximately 60% of astrocytes in the pre-frontal cortex positive for GFAB were also found to be C3 positive, indicating that A1 astrocytes promote neurodegenerative diseases [14]. Other studies have also mentioned that reactive astrocytes contribute to the progression of trauma and in the chronic pain that follows [19]. Nevertheless, the protective effect of reactive astrocytes on pain is still not completely understood, and it is unclear whether they play a dual role in chronic pain. We can hypothesize that A1 reactive astrocytes may be involved in chronic pain by releasing molecules such as inflammatory cytokines, chemokines and intracellular kinases [20].

The A2 astrocytes are induced by ischemia and promote neuronal viability as well as tissue regeneration. The A2 astrocytes contain specific markers such as S100A10 belonging to the S100 protein family [21]. These cells promote tissue regeneration and neuroprotection by secreting several growth factors. Expression of the S100A10 gene in the A2 astrocytes is essential due to the fact that this expression leads to proliferation, cell membrane repair, and inhibition of apoptosis. In addition, these astrocytes increase the expression of an anti-inflammatory cytokine called TGF, which participates in the process of synaptogenesis and induces neuroprotection [22]. The active astrocytes can regenerate the damaged blood-brain barrier, limiting the penetration of peripheral leukocytes [23]. Hence, the favorable effects of reactive astrocytes after the injury can be attributed to the A2 reactive astrocytes, which may delay or even impede the progression of chronic pain.

### 1.4 A1 to A2 reactive astrocyte transformation

To date, several treatment strategies have been developed to inhibit the activation of astrocytes. Intrathecal administration of astrocyte inhibitors, such as Valerine, Fluorocitrate, and L-1-amino-hexanedioic acid, effectively reverses mechanical allodynia, reducing abnormal pain and hyperalgesia in the pattern of pathological pain [[24], [25], [26], [27]]. However, reactive astrocytes also play an essential role during tissue regeneration of the scarred central nervous system (CNS) and have positive effects on the healing process following SCI via the STAT3 signaling pathway [28]. In another study, the deletion of the STAT3 gene of the astrocytes in a mouse model inhibited astrogliosis as well as increased inflammatory factor penetration and the post-SCI enlargement of the injured area, which directly points to the dual role of the presence or absence of astrocytes [29]. Therefore, specific inhibition of A1 reactive astrocytes may be a potential therapeutic target with more accurate effects and fewer side effects than the direct use of astrocyte inhibitors.

### 1.5 NeuroD1 factor role after nervous system injury

**Fig. 2:**
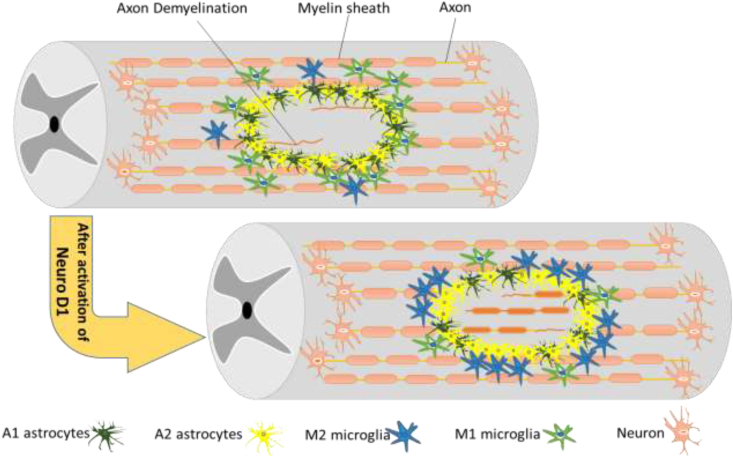
Activation of Neuro D1 has effects on the microenvironment of injured neural tissue through: 1. generation of new neurons 2. reduction of toxic A1 astrocytes (increase in A2 astrocyte activity) 3. attenuation of toxic M1 microglia, and 4. repair of blood vessels and BBB integrity.

Chen et al. have shown that the transformation of glial cells into neurons restores and rebalances the count of neurons and glial cells, causing the return of glial scars to the nerve tissue [30]. NeuroD1 is an endogenous neural transcription factor (Fig. 2). It has been shown that when NeuroD1 is expressed by reactive astrocytes, the A1 reactive astrocytes can become low-risk astrocytes and glial scar-forming astrocytes can also convert to neurons [[30], [31]]. In another study, a mouse model of Alzheimer's disease showed that the reactive astrocytes became functional neurons following the expression of NeuroD1. The same phenomenon was observed in human cortical astrocytes in an in vitro study [32]. A similar study showed that when NeuroD1 is highly expressed in astrocytes, new neurons formed by the reactive astrocytes recover 40% of the neurons lost in ischemic injury and also regenerate motor and memory deficits (Yuchen Chen et al., 2018). These studies show that the A1 astrocytes have the potential to convert to the A2 astrocytes and even to naive astrocytes. If the A1 astrocytes are involved in initiating chronic pain, promoting the process of converting the A1 astrocytes to the A2 astrocytes or even to naive astrocytes could therefore be a potential therapeutic strategy for pain relief.

Blocking upstream molecules or downstream targets of the A1 reactive astrocytes may also be an effective therapeutic approach. Research has shown that the active microglia, by secreting substances such as Il-1α, TNF, and C1q, convert the naive astrocytes to A1 astrocytes [[14], [33]]. Research has demonstrated that the MFG-E8 regulates the process of A1/A2 astrocyte conversion through upregulation of PI3K-Akt pathways and downregulation of NF-κB pathways in the culture medium [22]. Therefore, specific inhibition of these signaling molecules may reduce chronic pain. Moreover, *in vivo* A1/A2 astrocyte conversion is currently not well understood and needs further study.

## 2 Oligodendrocytes

The oligodendrocytes are the main source of myelin production in the CNS. These cells are prone to the most damage after SCI, which leads to extensive demyelination in the neurons of the affected area, which in turn increases the severity of the damage to the patient [34]. Since the development of cell transplantation, especially stem cell transplantation, as a promising post-SCI treatment strategy in recent years, the use of oligodendrocyte precursor cells (OPCs) has also received much attention for SCI therapy [35]. In the early stages after SCI, the proliferation of OPCs occurs close to the lesion site, but at a later time post-injury, proliferation can also be found in the spared white matter [[36], [37], [38]].

### 2.1 Differentiation of oligodendrocytes into their progenitor cells after SCI

Various studies have shown that factors, such as TNF α and IL-1β, as well as oxidative and ischemic stress in the chronic phase after SCI, cause a severe decrease in the count of oligodendrocytes. Apoptosis in these cells begins 15 min after the injury and continues for up to 3 weeks thereafter [[39], [40], [41], [42], [43], [138]]. Also, another factor called autophagy occurs at the site of injury, which begins 21 days after injury and occurs more frequently in oligodendrocytes than astrocytes. Becline-1 expression induces autophagy in the lesion site [44,45]. The differentiation of oligodendrocytes into their progenitor cells is regulated by molecules such as IGF1, FGF2, and CNTF. A number of studies have shown that the OPCs not only can produce A1 and A2 astrocytes [34,46,47], but also can regenerate myelin after injury [48]. In addition, the ability to transform these cells into neurons has also been recently confirmed [49]. These cells are present in the CNS in both white matter and grey matter. After SCI, the proliferation rate of OPCs at the end of the first day will increase significantly, and this increase will continue for one week. The cell counts will remain high for about 4 weeks. In general, two types of OPCs may be seen around the site of injury, round-shape OPCs and short thick processes [50].

There are at least two sources of oligodendrocytes in the adult brain: Subventricular Zone (SVZ) progenitors and NG2 positive and PDGFα R-positive oligodendrocytes scattered throughout the nervous system [[51], [52], [53], [54]]. These cells account for 5–8% of the cells in the CNS and are located in the optic nerve, motor cortex, corpus callosum, and cerebellum and provide a source for oligodendrocyte replacement in adults [55]. Interestingly, these cells proliferate in adults with very little growth to keep their hemostatic levels constant, but their rate of proliferation is never the same as at the time of development or the time of injury. Oligodendrocytes grow 0.3% per year and replace oligodendrocytes even in all adults [56]. All of this evidence suggests that myelination is not limited to the developmental period and childhood, but continues into adulthood [34].

A recent study showed that demyelinating conditions (such as injury) cause the activation of mature OPCs and phenotypically return to fetal OPCs, producing cytokine IL1β and chemokine CCL2, increasing the OPC convergence and motility, and promoting their re-accumulation at the site of demyelination. This study showed that the OPCs are able to modulate post-injury inflammation and promote its regeneration [57]. Thus, one of the therapeutic targets could be the activation of oligodendrocytes at the site of injury and reverting their phenotype to neonatal oligodendrocytes via cytokines (such as IL1β) and chemokines (such as CCL2).

There are many factors involved in regulating differentiation or increasing the count of OPCs after injury: (1) Growth factors such as FGF-2, PDGF-A, and CNTF [[58], [59], [60], [61], [62], [63], [64]]. (2) Neurotrophins such as BDNF and NT-3 [[65], [66], [67]]. (3) Chemokines and cytokines such as CXCL12 (also known as SDF-1), CXCL1, LIF and IL-17A [[68], [69], [70], [71], [72], [73], [74], [75], [76]]. (4) Transcription factors such as OLIG1 and HMG family such as SOX5 and SOX6 [[77], [78], [79], [80]]. IL-17A activates the ERK1/2 pathway and converts OPCs into mature oligodendrocytes. However, this effect occurs through association with IL-1β, which itself is known to protect OPCs and their differentiators. Unlike the IL-17A, the IL-1β blocks the oligodendrocytes in the cell cycle and retards their mitotic potency [81]. Therefore, it is interesting to study the synergistic effects of elevated IL-17A and IL-1 levels after injury [73].

### 2.2 NG2+ cells and their role after CNS injury

Glial progenitor cells that express the chondroitin sulfate proteoglycan NG2, named NG2+ cells, compose the majority of proliferating cells in the adult CNS [82]. These cells more than any other cell type in the brain have been given many names: polydendrocytes; NG2 progenitor cells; synantocytes; NG2 cells; or more often, oligodendrocyte progenitor cells [83].

NG2+ OPCs are responsive to several different types of nerve injury [48], including demyelination, traumatic injury to the CNS, and chronic neurodegenerative diseases [[84], [85], [86], [87]]. Furthermore, uncontrolled growth of these progenitors leads to tumor formation [88], and recent studies have suggested that NG2+ cells are likely to be a cell of origin for certain forms of glioma [89,90], highlighting the importance of understanding how the proliferation of these cells is controlled *in vivo*.

It has been demonstrated that the NG2 - positive cells play a role in the formation and elimination of glial scars, suggesting that these progenitor cells are capable of detecting CNS injury and improving tissue regeneration [91]. After tissue injury, NG2/PDGFαR progenitor cells differentiate into oligodendrocytes and can induce remyelination in axons [92]. However, in patients who suffer from multiple sclerosis, changes in the CNS environment occur and cause OPCs to lose their ability to respond to damaged myelin over time and to limit their myelination capacity [139]. It is thought that the OPCs need to be activated for remyelination. These progenitor cells respond to growth factors, mitogens, chemokines, and cytokines, which increase their proliferation and motility toward the injury site as well as increase the expression of the oligodendrocyte differentiation gene.

## 3 Astrocyte/ OPCs interaction after injury

### 3.1 Effects of astrocytes on oligodendrocytes after injury

Astrocytes, after an injury caused by various mechanisms, change to activated and reactive types and can affect the process of differentiation of oligodendrocytes through the secretion of different substances, which ultimately influences the myelination process. Some factors that have an excitatory effect on the myelination process and secreted by astrocytes include the following: factor-like protein (LIF), neuregulin-1 (NRG1), gamma-secretase (GS), ciliary neurotrophic factor (CNTF), insulin-like growth factor 1 (IGF-1), osteopontin (OPN), neurotrophin-3 (NT3) [[93], [94], [95], [96], [97], [98], [99]].

## 4 Factors secreted by astrocytes and enhancement of remyelination

In the cuprizone model (a model for demyelination), the expression of TNFR2 in the astrocytes leads to the expression of autocrine CXCL12 (which acts as a CXCR4 receptor on oligodendrocyte progenitor cells), which eventually leads to the proliferation and differentiation of OPCs [72]. The CNTF has been found in the astrocytes (both active and reactive) at the site of injury as well as around SCI, causing the regulation of FGF2 production in astrocytes in the early stages of remyelination, indicating that the CNTF is an important cytokine in diseases associated with demyelination [93]. Interestingly, in two studies of the demyelination model in the SCI, white matter in the spinal cord revealed that the absence of astrocytes at the site of injury reduced oligodendrocyte-mediated remyelination and increased remyelination by Schwann cells [100,101]. Taken together, these results suggest that astrocyte-free regions at the site of injury either contain inhibitory signals that block the final OPC differentiation or lack the signals necessary for the OPC to make the final differentiation.

## 5 Astrocyte-derived inhibitors causing demyelination and reducing remyelination

Initially, astrocytes were described as cells that inhibit the differentiation of oligodendrocytes, especially at the time of glial scar formation. The astrocytes that are present in the glial scar inhibit both myelination and remyelination through upregulation or downregulation of related factors and bioactive molecules such as PDGF, FGF2, and tenascin C, BMP2/4, and hyaluronan. Hyaluronan is a glycosaminoglycan (GAG) that interacts with CD44 (a receptor expressed in OPCs) and in some situations like lysolecithin-induced demyelination in white matter, this interaction impairs the remyelination process. One reason for this defect is that OPCs could not differentiate into myelin-producing cells at the site of injury, which is high in hyaluronan quantity (hyaluronan at the site of injury reduces the process of differentiation of OPCs into myelin-producing cells). Another study showed that the introduction of hyaluronan into the culture medium inhibited OPC differentiation [102].

Astrocyte-derived endothelin-1 (ET-1) is also known to be an inhibitor of the differentiation process of OPCs through increased expression of Jagged 1 that causes Notch activation on OPCs [103]. Researchers have shown that the level of myelination was higher after transplantation of neonatal OPCs to the demyelinated site without astrocytes compared to the injury site with astrocytes [104] (influence of astrocytes on remyelination at the site of injury). Another study of SCI showed that preventing astrocyte glial scars significantly reduces the stimulation of axon regeneration [105]. Although no data were available on myelin in this study, they indicated a positive role for astrocytes in the regeneration process. Overall, these studies indicate that glial cell interactions in the myelination process are largely influenced by the surrounding environment. Depending on what stage of astrocytes is activated, they can exert their own stimulatory or inhibitory effects on the development process of oligodendrocytes. In addition to the different types of astrocytes, the distance of these cells to the site of injury must also be considered, as relatively small changes in the environment may have different effects on the behavior of oligodendrocytes. Nash et al. [106] proposed the hypothesis that, whereas the astrocytes that are distal to the activated cells are more likely to play a role in regeneration through the secretion of growth factors and cytokines, the astrocytes at the site of injury show more reaction (reactive astrocytes) and may impede the remyelination process. The process of activation and the elevation of astrocytes present in the distal part of the injury site while inhibiting the activity of astrocytes at the site of injury must be taken into account.

### 5.1 Relation between ependymal cells and OPCs

Interestingly, recent studies have demonstrated that the ependymal cells around the spinal canal and at the site of the injury can be a source of oligodendrocytes. Although the level of the proliferative activity in ependymal cells is only one-tenth of that of OPCs under normal conditions, the ependymal cells proliferate twice as much as OPCs under injury. Surprisingly, some of these cells eventually differentiate into functional oligodendrocytes [107]. A new study has shown a cholinergic increase in the proliferation of ependymal cells, which has led to increased oligodendrocyte markers in the spinal cord tissue [108]. Other studies have also indicated that ependymal cells differentiate into astrocytes and oligodendrocytes after SCI [[107], [108], [109]]. This gives rise to an idea that proposes the production of oligodendrocytes by ependymal cells. One research idea could be the investigation of ependymal cells and their role in the differentiation of oligodendrocytes after injury. Given the evidence that these cells can be differentiated into oligodendrocytes, they can be used as a therapeutic approach in combination with other methods in regenerative medicine.

### 5.2 Macrophage and microglia

Macrophages are a major cell type of the immune system and constitute both resident tissue and circulating monocyte-derived subsets. Here, we have focused on microglia, resident macrophage in CNS, and the first line of defense against pathological circumstances and nervous tissue damages. As a matter of fact, these cells have crucial rules in the regulation of CNS disorders [58]. Thus, most current treatment strategies focus on targeting this subset. To our mind, a better understanding of the biology of macrophages gives birth to a discussion of new routes for therapeutic intervention.

### 5.3 Timespan (time-course) and source of macrophages after SCI

#### 5.3.1 Splenic versus bone marrow-derived macrophages

**Fig. 3:**
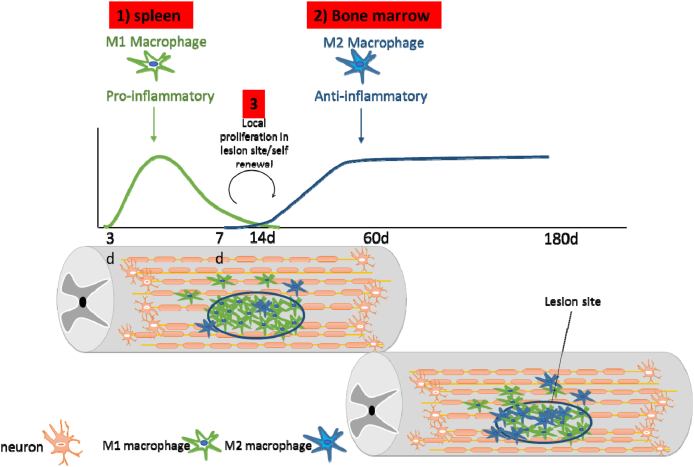
The three main sources of Macrophage cells after spinal cord injury (SCI) are represented in numeric format. The first wave of Macrophage influx occurs on day 3 and reaches a peak on day 7. The second wave begins on day 14 and peaks again on day 60. The main source of first wave macrophages is the spleen and mostly contains M1 Macrophage and the second wave of macrophages could be from either the bone marrow or from a self-renewing source at the injury sites and contains both M1 and M2 macrophages.

After SCI, the injured tissue secretes cytokines and chemokines into the circulatory system, recruiting monocytes to the injury site. These monocytes become macrophages in several ways upon reaching the injury site. These macrophages play various roles in wound healing. The first wave begins on day 3 after injury and reaches its maximum level on day 7 (Fig. 3). After a slight decrease, the second wave initiates on day 14 and reaches its maximum level on day 60 and remains at that level until day 180 [110,111]. This form of an increase in macrophage counts is found in humans, although it is unclear whether the second wave of elevation is also seen in humans [112,113]. Until now, bone marrow stem cells were known only as a source for monocytes, but recently researchers have found that the spleen also acts as a source of monocytes during injury [112]. A study showed a significant decrease in macrophage counts at the SCI site after splenectomy [114]. The macrophages remain at the site of injury for a long time, but the life span of monocytes is 1–3 days, and the macrophages produced by these monocytes have a life span of several weeks [115]. Therefore, the question arises as to how macrophages are replaced regularly during SCI or where they originate in these types of injuries. The old answer to this question is that since circulating monocytes eventually become macrophages, the emergence of new macrophages requires more monocyte influx. Although the evidence suggests that the local proliferation of macrophages at the site of injury affects their population growth, the local proliferation of macrophages (at the site of SCI) needs further research.

### 5.4 Microglia, resident macrophage in CNS

**Fig. 4:**
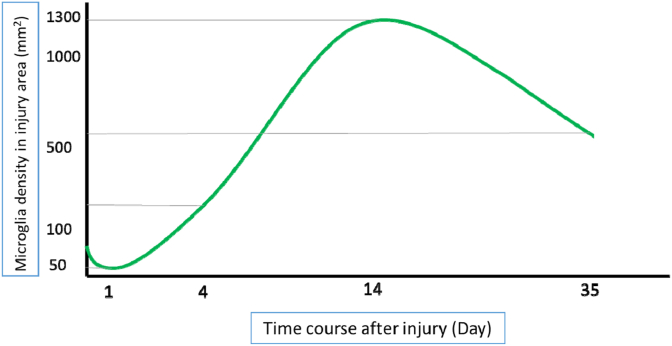
Time-course profiles of microglial activation after SCI.

Microglia make up 5–10% of all central nervous system cells. It has been previously shown that in a healthy condition, the microglial population in the brain remains constant, meaning that there is a balance between cell death and cell proliferation [116]. However, there was little information about the microglial population in the spinal cord and how these cells react following injury. The dynamic response of microglia cells to SCI has been confirmed in many studies [8,[116], [117], [118], [119]]. Lacroix et al. in a study have shown that microglia are immediately recruited in the injury site in the spinal cord. In terms of proliferation, they scrutinized a specific antibody of microglia and found that the number of microglia decreased slightly 24 h after the lesion. However, the population changed and there was a significant increase since then, showing a four-fold increase at day 4, continuing to increase at day 14, reaching up to 10 times compared to day 1 (Fig. 4) [116,118]. Abdanipour et al. reported that microglia profile activation reached the highest level at day 2 after SCI [1]. Another study on a mouse model of SCI observed that microglia had reached up to a peak on day 7 and then was reduced at day 14 [121]. All in all, it is obvious that microglial cells are highly dynamic and proliferate extensively during the first two weeks after SCI.

There are two characteristics of microglia that separate this cell type from other tissue-resident macrophages—limitations on prenatal origin, and their capacity for regeneration and longevity [122]. After birth, the macrophage cells residing in each tissue, as well as the monocytes existing in the circulation, are continuously replaced by myeloid cells, which is not the case with microglia cells [123,124]. Activation of microglia is the first response of the nervous system after SCI. This presence is provided by two sources, which include microglia at the site of injury and microglia recruited to the site of injury through blood flow. Extensive research has been conducted on the activity of microglia after SCI, which points to the dual role of these cells. Researchers have found that microglia activity is like a double-edged sword that can both be useful for healing and remodeling after injury and exacerbating the extent of post-injury damage [116].

### 5.5 Macrophage/microglia polarization

In terms of polarization, macrophages have been classically divided into M1 and M2 groups by Mills et al., in 2000 [125]. The M2 microglia, in turn, can be divided into three subsets, namely, alternative activation (M2a), alternative type II activation (M2b), and acquired deactivation (M2c) [[126], [127]]. However, scientists now recognize that M1 and M2 microglia are not perceptibly different from each other and that the polarization state is a continuum process between them [128]. Furthermore, the type of polarized activation of macrophages depends on many factors such as the microenvironment, stage, course, and severity of the posttraumatic process [129,130], and the M1/M2 paradigm can be variable in response to different stimuli [128]. It should be noted that many scientists place macrophages and microglia into the same cell population and make no distinction between them, and usually have been using pan markers to identify them.

### 5.6 Relation between M2/M1 macrophage ratio and repair after injury

Subsequent studies have shown that M1 macrophages produce pro-inflammatory cytokines and chemokines and kill their surrounding cells and inhibit cell proliferation, and in contrast M2 macrophages promote cell proliferation and tissue growth by secreting growth factors, neurotrophic factors and anti-inflammatory cytokines [131,132]. These results led the researchers to think that the switch of macrophage polarization to the M2 type would alleviate injury. Kigerl et al. [130], in the first study, showed that M1 macrophages accounted for most of the macrophages at the site of injury and that M2 macrophages had a temporary presence within the first 7 days after injury.

At transcriptional levels, IFN-α and LPS have been shown to be classical receptors in M1 macrophages [133]. The LPS receptor, TLR4, plays a key role in the degradation process at the injury site. The TNF and IL-1β cytokines are highly expressed after SCI [73,134]. The highest level of mRNA expression was in TNF 1 h after injury and IL-1β 12 h after injury [73]. In contrast, IL-4 and IL-10 are classical ligands for M2 macrophages. The IL-4 has a major effect on the M2 macrophage polarization by activating STAT6 [135]. The IL-10 also indirectly inhibits the expression of pro-inflammatory cytokines through the JAK1/STAT3 pathway [136].

The source of macrophages is another case that has been investigated so far. One possibility is that the injury site environment affects the monocytes recruited to the area and preferentially polarizes them to the M1 type. However, the effect of the injury environment on M1 macrophages is one of the features of SCI. This has led various researchers to focus on changing the environment to reduce the M1 macrophage counts. For example, the ChABC enzyme, by destroying CSPGs, causes M2 macrophages to form at the injury site [137].

At present, it is quite clear that macrophages play a key role in the wound healing process after SCI. However, some fundamental issues need to be addressed. The macrophages originate from splenic monocytes in acute conditions, but there appears to be an alternative source for chronic macrophages. Is there an endogenous source of macrophages that causes these cells to regenerate? Or are these macrophages derived from the circulation into the lesion area due to the continuous entry of bone marrow-derived monocytes? These are the issues that need to be addressed. Although M1/M2 classifications are conceptually useful for macrophages, their polarization steps are still unclear for *in vivo* conditions.

## 6 Conclusion

Glial cells in the nervous system can play both beneficial and detrimental roles after SCI, some of which are summarized in this article. With this in mind, the most comprehensive treatment strategies for better recovery and improvement after SCI can include the following:

1) Increasing the A2/A1 astrocyte ratio,
2) Increasing the M2/M1 macrophage ratio, and
3) Increasing the differentiation power of OPCs cells and turning them into oligodendrocytes or even neurons. Obviously, applying a comprehensive strategy that covers all of these phenomena simultaneously can give a better response in terms of injury inhibition and trauma restriction. By studying these cells more closely and focusing more on their function, solutions can be highlighted that involve the beneficial roles of these cells in the nervous system, thereby taking the small but necessary step to helping the healing and repair process after spinal injury.

## Funding statement

Not available.

## Declaration of competing interest

I wish to confirm that there are no known conflicts of interest associated with this publication and there has been no significant financial support for this work that could have influenced its outcome.

I confirm that the manuscript has been read and approved by all named authors and that there are no other persons who satisfied the criteria for authorship but are not listed. I further confirm that the order of authors listed in the manuscript has been approved by all of authors.

I confirm that the authors have given due consideration to the protection of intellectual property associated with this work and that there are no impediments to publication, including the timing of publication, with respect to intellectual property. In so doing I confirm that we have followed the regulations of our institutions concerning intellectual property.
